# Evaluation of Subjects Experiencing Allergic Reactions to Non-Steroidal Anti-Inflammatory Drugs: Clinical Characteristics and Drugs Involved

**DOI:** 10.3389/fphar.2020.00503

**Published:** 2020-04-21

**Authors:** Natalia Pérez-Sánchez, Inmaculada Doña, Gador Bogas, María Salas, Almudena Testera, José A. Cornejo-García, María J. Torres

**Affiliations:** ^1^Allergy Unit, Malaga Regional University Hospital, Malaga, Spain; ^2^Departamento de Medicina, Universidad de Málaga, Malaga, Spain; ^3^Allergy Research Group, Instituto de Investigación Biomédica de Málaga-IBIMA, ARADyAL, Malaga, Spain; ^4^Nanostructures for Diagnosing and Treatment of Allergic Diseases Laboratory, Andalusian Center for Nanomedicine and Biotechnology-BIONAND, Malaga, Spain

**Keywords:** drug allergy, non-steroidal anti-inflammatory drugs, urticarial, anaphylaxis, clinical immunology

## Abstract

Non-steroidal anti-inflammatory drugs (NSAIDs), the most commonly prescribed and consumed medicines worldwide, are the main triggers of drug hypersensitivity reactions (DHRs). The underlying mechanisms of NSAID-DHRs may be related to COX-1 inhibition (cross-hypersensitivity reactions, CRs) or to immunological recognition (selective reactions, SRs), being the latter remarkably less studied. SRs include those usually appearing within the first hour after drug intake (single-NSAID-induced urticaria/angioedema or anaphylaxis, SNIUAA), and those usually occurring more than 24 h after (single-NSAID-induced delayed reactions, SNIDR). We have evaluated the largest series of patients with SRs, analyzing the number of episodes and drugs involved, the latency for reaction onset, the clinical entities, among other variables, as well as the value of available diagnostic methods. Globally, pyrazolones and arylpropionics were the most frequent culprits (39.3% and 37.3%, respectively). Pyrazolones were the most frequent triggers in SNIUAA and arylpropionics in SNIDR. Urticaria was the most common clinical entity in SNIUAA (42.4%) followed by anaphylaxis (33.3%); whereas SNIDR induced mostly fixed drug eruption (41.1%) and maculopapular exanthema (32.6%). The percentage of patients diagnosed by clinical history was higher in SNIUAA compared with SNIDR (62.7% versus 35.3%, *p* = 0.00015), whereas the percentage of those diagnosed by skin tests was higher in SNIDR than in SNIUAA (47.1% versus 22.8%, *p* = 0.00015). Drug provocation test with the culprit was performed in 67 SNIUAA (14.5%) and in 9 SNIDR (17.6%) patients. Our results may be of interest not only for allergologists but also for other clinicians dealing with these drugs, and can be useful for the correct identification of subjects experiencing DHRs to NSAIDs, and for avoiding mislabeling. Moreover, as NSAIDs are highly consumed worldwide, our results may be of interest for evaluating other populations exposed to these drugs.

## Introduction

Non-steroidal anti-inflammatory drugs (NSAIDs) are the most frequent triggers of drug hypersensitivity reactions (DHRs) (Doña et al., 2012; Aun et al., 2014; Blanca-Lopez et al., 2014; Jares et al., 2015). These DHRs are of great concern, as NSAIDs are the most commonly prescribed and consumed medicines worldwide (Rao and Knaus, 2008; Conaghan, 2012; Brune and Patrignani, 2015).

The latest classification of NSAID-induced hypersensitivity distinguishes five phenotypes (Kowalski et al., 2013), which can be grouped into two major categories based on their underlying mechanisms: i) non-allergic or cross-hypersensitivity reactions (CRs), and ii) allergic or selective reactions (SRs). CRs are induced by chemically distinct (non-related) drugs and do not require previous immunological recognition; SRs require this recognition to a single/group of drug(s), with subjects tolerating other chemically non-related NSAIDs, including strong COX-1 inhibitors. The latter includes immediate reactions, which occur in most patients up to 1 h after drug intake (single-NSAID-induced urticaria/angioedema or anaphylaxis; SNIUAA); and delayed reactions, which occur more than 24 h after drug intake (single-NSAID-induced delayed reactions; SNIDR).

Although the immunological mechanisms underlying these reactions are not completely understood, SNIUAA is thought to be mediated by specific IgE antibodies, despite the presence of such antibodies being only demonstrated for the pyrazolone derivative propyphenazone (Himly et al., 2003). In addition, the use of basophil activation test (BAT) has supported an IgE mechanism for SNIUAA by metamizole (Gomez et al., 2009; Ariza et al., 2014). However, no experimental evidence exists for an IgE-dependent mechanism for SNIUAA induced by other related drugs such as diclofenac (Harrer et al., 2010). Regarding SNIDR, a T cell-mediated mechanism has been proposed (Kowalski et al., 2013).

The difficulties on establishing the molecular basis of the underlying mechanisms hamper the development of *in vitro* diagnostic tests. Moreover, skin tests (STs) are only useful for pyrazolones and paracetamol, with low sensitivity (Kowalski et al., 1999; de Paramo et al., 2000; Brockow et al., 2002; Gomez et al., 2009; Blanca-Lopez et al., 2016). Finally, drug provocation test (DPT), the gold standard to confirm diagnosis, is a not risk-free procedure (Aberer et al., 2003). These facts have important clinical implications, as patients with SRs may unnecessarily avoid all NSAIDs when only a specific NSAID or a group of chemically related NSAIDs trigger such reactions.

Although there is a lack of epidemiological studies on NSAIDs-hypersensitivity, the relative contribution of CRs and SRs seems to vary among countries (Doña et al., 2011; Chaudhry et al., 2012; Demir et al., 2015). Most studies of DHRs to NSAIDs have focused on CRs and large series of cases confirmed as SRs to NSAIDs have not been globally analyzed (Doña et al., 2019). In this study, we have evaluated a large group of patients suffering from SRs to NSAIDs. We have focused on different variables, including the number of episodes and NSAIDs involved, the latency for reaction onset, the clinical entities, and the comorbidities associated. We also aimed to assess the value of the available methods for achieving the diagnosis of SRs to NSAIDs.

## Methods

### Patients Selection

**Figure 1:**
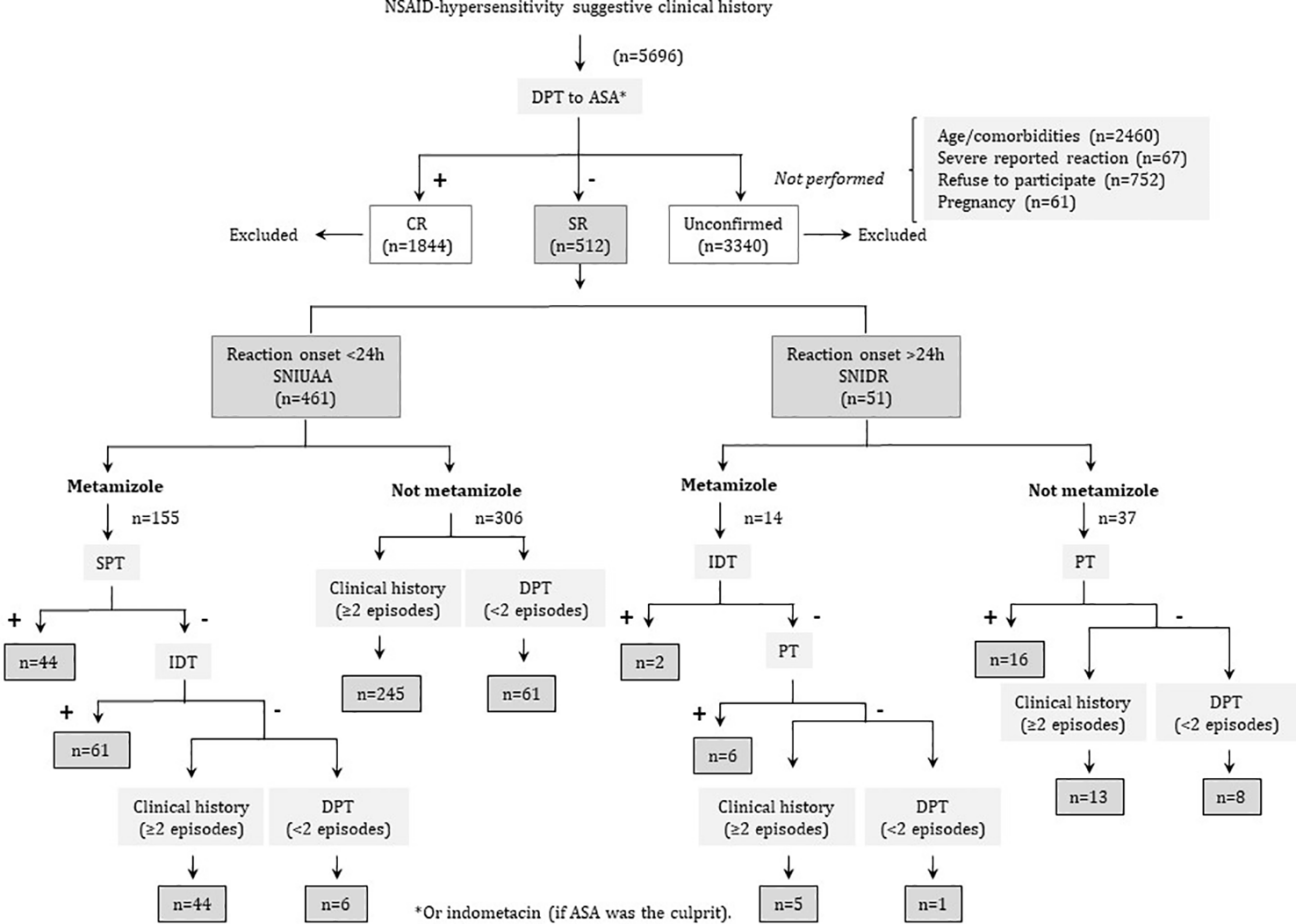
Clinical algorithm for patients’ diagnosis.

Patients with a suggestive clinical history of DHR to NSAIDs were prospectively evaluated from 2011 until 2019 in the Allergy Unit from the Malaga Regional University Hospital following a common protocol, slightly modified from the one of Doña et al. (Doña et al., 2011) (**Figure 1**).

Those cases with a confirmed diagnosis of SRs and older than 14 years were finally included in this study, whereas those with a confirmed diagnosis of CRs were not considered. We further excluded pregnant or breastfeeding patients, those taking β-blockers or ACE inhibitors, or those with contraindications to epinephrine administration, patients who had acute infections and/or underlying cardiac, hepatic or renal diseases that contraindicated DPT, and those with psychosomatic disorders.

This study was performed according to the principles of the Declaration of Helsinki, and approved by the local ethics committee. All patients were orally informed about the study and signed the corresponding informed consent.

### Protocol

Tolerance to acetylsalicylic acid (ASA) or indometacin (if ASA was the culprit) was verified by DPT. If subjects tolerated ASA/indometacin in DPT, they were considered as having either SNIUAA (when symptoms appeared <24 h after NSAID administration), or SNIDR (when symptoms appeared after 24 h or more).

For SNIUAA, when metamizole was involved, STs were performed as described previously (Blanca-Lopez et al., 2016). If positive, the patients were confirmed as presenting SNIUAA to metamizole, whereas if STs were negative we took into account the number of episodes. The number of episodes was also taken into account when metamizole was not the culprit. If patients had at least 2 episodes, they were diagnosed as SNIUAA, but if they experienced only one episode, a positive DPT with the culprit was required to confirm diagnosis. However, in those cases in which DPT was contraindicated (as described above) or in which severe reactions such as anaphylactic shock were reported, DPT was not performed, and patients were excluded from the study (**Figure 1**).

For SNIDR, STs with the culprit were performed also as described (Blanca-Lopez et al., 2016). If results were positive, patients were confirmed as having SNIDR. If negative, we considered the number of episodes suffered after NSAID intake: with at least 2 episodes they were diagnosed as SNIDR; however, a positive DPT with the culprit was required to confirm diagnosis when only one episode was reported. As above, if patients showed some contraindications for DPT or presented severe reactions, like Stevens-Johnson syndrome/toxic epidermal necrolysis or acute exanthematic pustulosis, this procedure was not performed and, consequently, patients were excluded from the study (**Figure 1**).

### Skin Testing

For SNIUAA to metamizole, skin prick testing (SPT) and intradermal testing (IDT) were performed using 400 and at 40 mg/ml, respectively, as described (Blanca-Lopez et al., 2016). In patients with severe reactions, IDT was carried out with dilutions from 1/10 to 1/100. An increase in the diameter of the wheal area of at least 3 mm developing 20 min after testing was considered a positive result. SPT and IDT were not performed with other drugs as their systematic use for *in vivo* diagnosis is not recommended (Brockow et al., 2002; Kowalski et al., 2013; Ortega et al., 2014).

For SNIDR, patch tests (PTs) with the culprit were performed as described (Brockow et al., 2002). In those cases in which metamizole was the culprit, IDT with delayed-reading was also performed (Kowalski et al., 1999; Blanca-Lopez et al., 2016).

### Drug Provocation Test

This procedure was carried out in a single blind manner. On the first day, placebo capsules were administered at different times, and on the second day (at least 1 week later) increasing doses of NSAIDs were administered orally at 90-min intervals, up to a total of two to five administrations depending on the drug (**Table 1**). If cutaneous and/or respiratory symptoms or alterations in vital signs appeared, DPT was stopped and symptoms were evaluated and treated. If the drug was tolerated, a therapeutic course of two days was performed 24 h afterward. All drugs were provided in opaque capsules prepared by the hospital pharmacy service.

**Table 1:** Doses of NSAIDs used in DPT.

Drug	Doses used in DPT (90-min intervals) (mg)	
	1st day	2nd day
Metamizole	5, 15, 50	70, 70, 150, 300
Dexketoprofen	1, 5	5, 10, 10
Ibuprofeno	5, 20, 50	75, 75, 150, 300
Ketoprofen	5, 10	12.5, 12.5, 25
Naproxen	5, 25, 50	75, 75, 150, 250
Diclofenac	5, 10	12.5, 12.5, 25
ASA	5, 20, 50, 50	100, 200, 200
Paracetamol	20, 30, 50, 100	150, 150, 200, 500
Lornoxicam	1, 1	2, 2, 4
Piroxicam	2.5, 2.5	5, 5, 10
Celecoxib	5, 15, 30	25, 25, 50, 100
Lisine clonixinate	5, 10, 20	31.25, 31.25, 62.5
Nabumetone	20, 30, 50, 100	150, 150, 200, 500

ASA, acetylsalicylic acid; DPT, provocation test.

### Atopic Status

This was assessed by SPT using a battery of 8 common inhalant allergens, including pollens, house dust mites, molds, and animal dander (ALK, Madrid, Spain). Histamine hydrochloride (10 mg/ml) and phenolated glycerol saline were used as positive and negative controls, respectively. Patients were requested to stop taking any medications that contained antihistamine at least 8 days before SPT. A positive SPT response was defined by the development of a wheal with a diameter of at least 3 mm to one or more of these allergens, and consequently patient considered atopic.

### Statistical Analysis

The chi-squared or the Fisher tests were used to analyze differences in nominal variables between groups, and the Mann-Whitney test for quantitative variables. All reported p-values represent two-tailed tests, with values <0.05 considered statistically significant.

## Results

### Subjects Evaluated

In the Allergy Service of the Malaga Regional University Hospital, a total of 5696 patients with a suggestive history of hypersensitivity to NSAIDs were evaluated. Among them, 512 (9%) were confirmed as SRs. The remaining 5184 patients were excluded from the study either because they were confirmed as CRs (1844, 35.6%) or because diagnosis was not achieved (3340, 64.4%). From the latter, 2460 patients did not undergo DPT to ASA (47.5%) due to age or comorbidities, 67 patients did not undergo DPT to the culprit (1.3%) due to the severity of the reported reaction, 61 were excluded due to pregnancy (1.2%), and 752 refused to participate in the study (14.5%).

Within the SR group, 461 patients were confirmed as SNIUAA (90%) and 51 as SNIDR (10%). Females were more frequently affected than males (342; 66.8%), and the median age at diagnosis was 43 years (IR: 31.5–54). No differences between SNIUAA and SNIDR were found regarding sex and age.

Two hundred fifty-seven patients were atopic (50.2%), being the most commonly involved allergens *Dermatophagoides pteronyssinuss* (150; 29.3%), *Olea europaea* (141; 27.5%), and *Lolium perenne* (80; 15.6%). Underlying rhinitis was present in 104 patients (20.3%), asthma in 51 (10%), food allergy in 16 (3.1%), and chronic spontaneous urticaria in 15 (2.9%). No differences were found between SNIUAA and SNIDR for any of these variables (**Table 2**).

**Table 2:** Clinical characteristics of patients included in the study.

		Total (N = 512)	SNIUAA (n = 461)	SNIDR (n = 51)	p
Sex (F/M)		342/170	309/152	33/18	NS
Age (years), median (IR)		43 (31.3–54)	42 (30–53)	45 (36–57)	NS
Rhinitis, n (%)		104 (20.3)	93 (20.2)	11 (21.6)	NS
Asthma, n (%)		51 (10)	46 (10)	5 (10)	NS
Food allergy, n (%)		16 (3.1)	15 (3.3)	1 (2)	NS
Chronic urticaria, n (%)		15 (2.9)	15 (3.3)	–	NA
Atopy, n (%)		257 (50.2)	236 (51.2)	21 (41.2)	NS
Sensitization, n (%)	Grass	80 (15.6)	72 (15.6)	8 (15.7)	NS
	Olive	141 (27.5)	129 (28)	12 (23.5)	NS
	*D. pteronyssinus*	150 (29.3)	134 (29.1)	16 (31.4)	NS
	Parietaria	39 (7.6)	33 (7.2)	6 (11.8)	NS
	Alternaria	33 (6.4)	62 (13.4)	1 (2)	NS
	Dog dander	65 (12.7)	58 (12.6)	7 (13.7)	NS
	Cat dander	80 (15.6)	71 (15.4)	9 (17.6)	NS
	Pru p 3	53 (10.4)	48 (10.4)	5 (10)	NS

NA, not applicable; NS, not significant.

### Diagnostic Methods

Patients reporting at least 2 episodes with the same drug were diagnosed by their clinical history (307; 60%). When this condition was not fulfilled, diagnosis was achieved either by a positive result in ST (129; 25.2%) or in DPT (76; 14.8%) with the culprit. The time interval between the last reaction and the first evaluation in our Allergy unit was 12 months (IR: 6–36), with no differences between SNIUAA and SNIDR.

The percentage of patients diagnosed by clinical history was higher in SNIUAA compared with SNIDR (62.7% versus 35.3%, *p* = 0.00015), and the percentage of patients diagnosed by STs was higher in SNIDR than in SNIUAA (47.1% versus 22.8%, *p* = 0.00015). In SNIUAA, STs were performed only when metamizole was the culprit (n = 155), being positive in 105 (67.7%): 44 by SPT (28.4%) and 61 by IDT (55%). The time interval between the last reaction and the allergological work-up was shorter in patients with positive STs (median: 6 months, IR: 6–12, versus median: 12 months, IR: 6–24; *p* = 0.001). Anaphylaxis was most frequent in patients with a positive SPT compared with those with a negative SPT (n = 33, 75%, versus n = 51, 53.7%; *p* = 0.03), and in patients with a positive IDT (n = 33, 75%, versus n = 31, 50.82%; *p* = 0.02). No differences were found regarding sex, age, comorbidities, other symptoms reported, and the latency period (data not shown).

In SNIDR, delayed-reading IDTs with metamizole were performed in 4 cases, being positive in 2 patients who reported MPE. PTs were performed in 31 patients, being positive in 22 (71%): ketoprofen (n = 6), metamizole (n = 5), naproxen (n = 2), etofenomate (n = 4), diclofenac (n = 3), deketoprofen (n = 1), and celecoxib (n = 1). Patients with positive PTs reported MPE (n = 7), FDE (n = 7), contact eczema (n = 4), and four patients reported urticaria, AE, bullous exanthema, and SJS/TEN (n = 1 for each clinical entity).

DPT with the culprit was performed in 76 patients (14.8%): 67 SNIUAA (14.5%) and 9 SNIDR (17.6%). Regarding SNIUAA, DPT with metamizole was performed in 6 patients (all of them reported urticaria), which represents a 2.9% of cases in which pyrazolones were the culprit. In addition, DPT was performed in 61 SNIUAA patients in whom pyrazolones were not involved (13.2%): ibuprofen (n = 21), paracetamol (n = 19), diclofenac (n = 8), ASA (n = 7), and dexketoprofen, naproxen, lornoxicam, piroxicam, nabumetone, and lysine clonixinate (n = 1 for each drug). In this group, reported clinical entities were urticaria (n = 45), AE (n = 14), and rhinitis (n = 2). Concerning SNIDR, DPT was performed with ibuprofen and ASA and metamizole (n = 2 for each), and with ketoprofen, diclofenac, metamizole, and piroxicam (n = 1 for each). From these patients, 4 reported MPE, 3 reported FDE, and 2 reported urticaria.

All patients were asked about other drugs taken together with the culprit NSAID or in the context of the reaction. In a low proportion of them (n = 42), antibiotics (mainly amoxicillin or amoxicillin-clavulanic) were taken simultaneously with the culprit NSAID or in the context of the reported reaction. All these patients were challenged with the specific antibiotic, and none of them developed a reaction. Moreover, patients reporting only one episode were also challenged with the culprit NSAID. In patients reporting more than one episode, only one episode was induced after the simultaneous intake of the NSAID and the antibiotic, whereas the remaining episodes were induced only after the intake of NSAID.

### Number of Episodes and Drugs Involved

Patients suffered a total of 1070 episodes (median 2; IR: 2–3 episodes), being 975 in SNIUAA patients (91.1%), and 95 in SNIDR patients (8.9%). Concerning the number of episodes per patient, 111 reported one episode (21.7%), 260 two episodes (50.8%), and 141 three or more episodes (27.5%), with no differences between SNIUAA and SNIDR.

Pyrazolones and arylpropionic acid derivatives were the most frequent culprits (420, 39.3%; and 399, 37.3%, respectively), followed by para-aminofenols (paracetamol) (102, 9.5%), arylacetic acid derivatives (88, 8.5%), and salicylates (40, 3.7%). Oxicams, fenamates (etofenamate), nicotinic acid derivatives (lysine clonixinate), selective COX-2 inhibitors (celecoxib), and naphthylalkanone (nabumetone) were less frequently involved (**Table 3**). Pyrazolones were the most frequent triggers in SNIUAA (393, 40.3% versus 27, 28.4%; *p* = 0.024) whereas arylpropionic acid derivatives in SNIDR (39, 41.1% versus 360, 36.9%; *p* > 0.05) (**Table 3**).

**Table 3:** NSAIDs involved in SRs included in this study.

		Total (N = 1070), n (%)	SNIUAA (n = 975), n (%)	SNIDR (n = 95), n (%)	p
*Pyrazolones*		420 (39.3)	393 (40.3)	27 (28.4)	0.024
	Metamizole	379 (35.4)	352 (36.1)	27 (28.4)	0.135
	Propyphenazone	41 (3.8)	41 (4.2)	–	NA
*Propionic derivatives*		399 (37.3)	360 (36.9)	39 (41.1)	0.427
	Ibuprofen	295 (27.6)	284 (29.1)	11 (11.6)	2e-4
	Naproxen	49 (4.6)	37 (3.8)	12 (12.6)	8e-5
	Dexketoprofen	34 (3.2)	29 (3)	5 (5.3)	0.225
	Ketoprofen	21 (2)	10 (1)	11 (11.6)	1.5e-6
*Paracetamol*		102 (9.5)	92 (9.4)	10 (10.5)	0.73
*Arylacetic derivatives*		88 (8.5)	81 (8.3)	7 (7.4)	0.75
	Diclofenac	80 (7.5)	74 (7.6)	6 (6.3)	0.652
	Aceclofenac	8 (0.7)	7 (0.7)	1 (1)	0.718
*ASA*		40 (3.7)	38 (3.9)	2 (2.1)	0.379
*Oxicams*		11 (1)	7 (0.7)	4 (4.2)	0.001
	Lornoxicam	1 (0.1)	1 (0.1)	–	NA
	Meloxicam	1 (0.1)	1 (0.1)	–	NA
	Piroxicam	9 (0.8)	5 (0.5)	4 (4.2)	0.0001
*Etofenomate*		7 (0.7)	2 (0.2)	5 (5.2)	4.6e-8
*Lisine clonixinate*		1 (0.1)	1 (0.1)	–	NA
*Celecoxib*		1 (0.1)	–	1 (0.1)	NA
*Nabumetone*		1 (0.1)		–	NA

ASA, acetylsalicylic acid; SNIDR, single-NSAID-induced delayed reactions; SNIUAA, single-NSAID-induced urticaria/angioedema or anaphylaxis; SRs, selective reactions; NA, not applicable.

Data describing the number of patients and pharmacological groups/drugs involved in SRs are depicted in **Supplementary Table S1**. Considering the number of patients instead of the number of episodes, most SNIUAA patients reacted to pyrazolones, mainly to metamizole, followed by propionic and arylacetic acid derivatives, and paracetamol. In SNIDR most patients reported reactions to propionic derivatives, followed by pyrazolones (**Supplementary Table S1**).

### Clinical Entities

Globally, urticaria was the most common clinical entity, accounting for 428 episodes (40%), followed by anaphylaxis (325; 30.7%) and angioedema (222; 20.7%). Although less frequent, other entities were fixed drug exanthema (FDE) and maculopapular exanthema (MPE), which appeared in 39 (3.6%) and 31 (2.9%) episodes, respectively. Exclusive respiratory airways symptoms, without other organ involvement, occurred in 16 episodes (1.5%). Urticaria was the most frequent clinical entity in SNIUAA (413; 42.4%) followed by anaphylaxis (325; 33.3%); whereas FDE was the most frequent in SNIDR (39; 41.1%), followed by MPE (31; 32.6%) (**Tables 4** and **5**).

**Table 4:** Clinical entities induced by each drug in SNIUAA.

		Anaphylaxis (n = 325), n (%)	AE (n = 221), n (%)	Urticaria (n = 413), n (%)	Asthma (n = 11), n (%)	Rhinitis (n = 5), n (%)	*p*
*Pyrazolones* (n = 393)		220 (56)	28 (7.1)	142 (36.1)	3 (0.8)	–	2e-5
	Metamizole (n = 352)	200 (56.8)	25 (7.1)	124 (35.2)	3 (0.9)	–	2e-5
	Propyphenazone (n = 41)	20 (48.8)	3 (7.3)	18 (43.9)	–	–	0.114
*Propionic derivatives* (n = 360)		58 (16.1)	150 (41.7)	143 (39.7)	4 (1.1)	5 (1.4)	2e-5
	Ibuprofen (n = 284)	43 (15.1)	121 (42.6)	111 (39.1)	4 (1.4)	5 (1.8)	2e-5
	Naproxen (n = 37)	7 (18.9)	17 (45.9)	13 (35.1)	–	–	0.02
	Dexketoprofen (n = 29)	8 (27.6)	9 (31)	12 (41.4)	–	–	0.73
	Ketoprofen (n = 10)	–	3 (2)	7 (7)0	–	–	0.118
*Arylacetic derivatives* (n = 81)		24 (29.6)	12 (14.8)	45 (55.6)	–	–	0.137
	Diclofenac (n = 74)	20 (27)	12 (16.2)	42 (56.8)	–	–	0.156
	Aceclofenac (n = 7)	4 (57.1)	–	3 (42.9)	–	–	0.375
*Paracetamol* (n = 92)		22 (23.9)	13 (14.1)	56 (60.9)	1 (1.1)	–	0.005
*ASA* (n = 38)		1 (2.6)	13 (34.2)	21 (55.3)	3 (7.9)	–	8e-6
*Oxicams* (n = 7)		–	5 (71.4)	2 (28.6)	–	–	0.04
	Piroxicam (n = 5)	–	3 (60)	2 (40)	–	–	0.174
	Lornoxicam (n = 1)	–	1 (100)	–	–	–	NA
	Meloxicam (n = 1)	–	1 (100)	–	–	–	NA
*Etofenomate* (n = 2)		–	–	2 (100)	–	–	NA
*Lysine clonixinate* (n = 1)		–	–	1 (100)	–	–	NA
*Nabumetone* (n = 1)		–	–	1 (100)	–	–	NA

AE, angioedema; ASA, acetylsalicylic acid; NA, not applicable.

**Table 5:** Clinical entities induced by each drug in SNIDR.

		AE (n = 1), n (%)	Urticaria (n = 15), n (%)	FDE (n = 39), n (%)	MPE (n = 31), n (%)	BE (n = 3), n (%)	SJS/TEN (n = 1), n (%)	CE (n = 5), n (%)	p
*Pyrazolones* (n = 27)		–	3 (11.1)	15 (55.6)	8 (29.6)	–	1 (3.7)	–	0.246
	Metamizole (n= 27)	–	3 (11.1)	15 (55.6)	8 (29.6)	–	1 (3.7)	–	0.246
*Propionic derivatives* (n = 39)		1 (2.6)	3 (7.7)	17 (43.6)	15 (38.5)	1 (2.6)	–	2 (5.1)	0.437
	Ibuprofen (n = 11)	–	2 (18.2)	3 (27.3)	6 (54.5)	–	–	–	0.692
	Naproxen (n = 12)	–	–	7 (58.3)	5 (41.7)	–	–	–	0.555
	Dexketoprofen (n = 5)	1 (20)	–	–	4 (80)	–	–	–	0.01
	Ketoprofen (n = 11)	–	1 (9.1)	7 (63.6)	–	1 (9.1)	–	2 (18.2)	0.02
*Arylacetic derivatives* (n = 7)		–	–	1 (14.3)	4 (57.1)	–	–	2 (28.6)	0.08
	Diclofenac (n = 6)	–	–	1 (16.7)	3 (50)	–	–	2 (33.3)	0.11
	Aceclofenac (n = 1)	–	–	–	1 (100)	–	–	–	NA
*Paracetamol* (n = 10)		–	6 (60)	4 (40)	–	–	–	–	0.008
*ASA* (n = 2)		–	–	–	2 (100)	–	–	–	NA
*Oxicams* (n = 4)		–	2 (50)	–	–	2 (50)	–	–	0.001
	Piroxicam (n = 4)	–	2 (50)	–	–	2 (50)	–	–	0.01
*Etofenomate* (n = 5)		–	1 (20)	2 (40)	1 (20)	–	–	1 (20)	0.543
*Celecoxib* (n = 1)		–	–	–	1 (100)	–	–	–	NA

AE, angioedema; ASA, acetylsalicylic acid; BE, bullous exanthema; CE, contact eczema; FDE, fixed drug exanthema; MPE, maculopapular exanthema; SJS/TEN, Stevens-Johnson syndrome/toxic epidermal necrolysis; NA, not applicable.

Concerning the specific culprit drug/drug group in SNIUAA, pyrazolones induced most frequently anaphylaxis (220; 56%; *p* = 0.00002); arylpropionic acid derivatives induced angioedema (150; 41.7%; *p* = 0.00002); and arylacetic acid derivatives, paracetamol and salicylates induced mainly urticaria (55.6%, 60.9%, and 55.3%, respectively) (**Table 4**). Regarding SNIDR, pyrazolones and arylpropionic acid derivatives induced most frequently FDE (55.6% and 43.6%, respectively), and arylacetic acid derivatives induced MPE (31; 32.6%) (**Table 5**).

Data concerning the number of SNIUAA and SNIDR patients and the specific clinical entities induced by each pharmacological group/specific drugs are depicted in **Supplementary Tables S2** and **S3**, respectively. In SNIUAA, the main triggers were pyrazolones and propionic acid derivatives (209 and 143 patients, respectively). Clinical entities induced by pyrazolones were mainly anaphylaxis and urticaria (57.4% and 37.7%, respectively), whereas propionic acid derivatives, mainly ibuprofen, induced both AE and urticaria (39.3% and 37.7%) (**Supplementary Table S2**). Urticaria was also the main entity induced by diclofenac and paracetamol (26 patients in both cases) (**Supplementary Table S2**). A total of 8 patients reported one episode of anaphylaxis and another episode of urticaria after metamizole intake, and one patient after ASA intake. Two patients reported one episode of urticaria and another one of AE induced by diclofenac. One patient reported one episode of asthma and another one of anaphylaxis after paracetamol, and one patient reported two episodes of urticaria after piroxicam and another one after meloxicam intake. Finally, one patient reported one episode of asthma and three of rhinitis after ibuprofen intake. Data concerning SNIDR were more heterogeneous (**Supplementary Table S3**).

### Time Interval Between Drug Intake and Onset of the Reaction (Latency Period)

Patients reacted a median of 30 min (IR: 10–60) after the NSAID intake, with this interval being shorter in SNIUAA than in SNIDR (30 min, IR: 10–60, versus 2880 min, IR: 1,440–4,320; *p* = 0.00000192).

As SNIDR episodes were limited, especially when compared with the number of SNIUAA episodes, we focused on the last entity to evaluate the relationship between time interval after drug intake-reaction onset and the specific culprit drug/drug group. Such interval was the shortest when pyrazolones were involved (10 min, IR: 5–30), and it was the longest when etofenomate was the culprit (420 min, IR: 270–570) (**Table 6**).

**Table 6:** Time interval between NSAID intake and reaction onset.

		Time interval drug intake-reaction onset	
		SNIUAA, min (IR)	SNIDR, days (IR)
*Pyrazolones*		10 (5–30)	1.4 (1–2.8)
	Metamizole	10 (5–30)	1.4 (1–2.8)
	Propyphenazone	10 (10–10)	–
*Propionic derivatives*		60 (30–120)	2 (2–4)
	Ibuprofen	60 (30–120)	4.5 (3.3–5.8)
	Naproxen	90 (60–165)	2.5 (1.8–3.3)
	Dexketoprofen	30 (25–90)	2 (2–2)
	Ketoprofen	240 (135–270)	2 (2–2)
*Arylacetic derivatives*		30 (11.3–105)	2 (2–2)
	Diclofenac	30 (15–120)	2 (2–2)
	Aceclofenac	10 (10–10)	2 (2–2)
*Paracetamol*		15 (10–60)	0.42 (0.33–0.42)
*ASA*		45 (30–60)	1 (1–1)
*Oxicam*		120 (65–150)	2 (2–2)
	Piroxicam	65 (37.5–92.5)	2 (2–2)
	Lornoxicam	180 (180–180)	–
	Meloxicam	65 (37.5–92.5)	–
*Etofenomate*		270 (270–270)	1 (1–1)
*Lysine clonixinate*		60 (30–120)	–
*Nabumetone*		360 (360–360)	–
*Celecoxib*		–	1 (1–1)
*p*		1.9e-6	0.427

ASA, acetylsalicylic acid; SNIDR, single-NSAID-induced delayed reaction; SNIUAA, single-NSAID-induced urticaria/angioedema or anaphylaxis.

Staying on SNIUAA, 187 episodes occurred within 1 to 6 h after NSAID intake (21.08%), with a time interval of 180 min (IR: 120–300 min): a total of 92 (49.19%) episodes were urticaria, 71 AE (38%); and 24 anaphylaxis (12.8%). Considering the drugs involved, in 112 episodes the culprit were arylpropionic acid derivatives (59.89%); pyrazolones in 25 (13.4%); arylacetic derivatives in 22 (11.8%); paracetamol in 16 (8.6%); ASA in 7 (3.7%); oxicams in 4 (2.1%); and nabumetone in 1 (0.5%).

## Discussion

NSAIDs are the most frequent triggers of DHRs, and it appears that the number of clinical entities that they can induce is greater than initially thought (Demir et al., 2015; Cousin et al., 2016). These reactions can be allergic (SRs) or non-allergic (CRs), with most studies focusing on the second group, in spite of SRs representing around a quarter of such reactions in some studies (Doña et al., 2011; Caimmi et al., 2012) or even more (Chaudhry et al., 2012; Demir et al., 2015). The relative contribution of CRs and SRs to DHRs to NSAIDs appears to vary depending on the country, with SRs accounting for around 20% of NSAID-hypersensitivity in Spain (Doña et al., 2011) and France (Caimmi et al., 2012); whereas these figures increase to over 40% in Australia (Chaudhry et al., 2012) and Turkey (Demir et al., 2015). From a clinical point of view, it is of great relevance to differentiate between CRs and SRs, as subjects mislabeled as CRs may unnecessarily avoid all NSAIDs whilst they do tolerate those that were not involved in the reactions (Kowalski et al., 2015; Doña et al., 2018).

Available information on SRs so far refers mainly to a specific drug/group of drugs (Blanca-Lopez et al., 2016), without considering SRs as a whole. In this study, we have focused on a large series of SRs, evaluating different clinical variables as well as the method used to achieve diagnosis. We first evaluated the participation of the different pharmacological group/specific drugs in SRs taking into account the number of episodes, obtaining similar results when we subsequently performed the analysis considering the number of patients.

We have confirmed that pyrazolone derivatives are the drugs most frequently involved in SNIUAA in Spain, as previously reported (Quiralte et al., 2007; Doña et al., 2011). We have also found that arylpropionic acid derivatives are the second most common cause of SNIUAA and the most important triggers in SNIDR. Differences between pyrazolones and arylpropionic acid derivatives differ with previous data from our group (44.6% versus 39.3% for pyrazolones, and 25.76% versus 37.4% for arylpropionic acid derivatives) (Doña et al., 2011). This variation in the pharmacological groups eliciting SRs may reflect a change in the pattern of NSAIDs consumption, as proposed (Doña et al., 2011; Doña et al., 2014). In fact, in countries where pyrazolones are not prescribed, arylpropionic acid derivatives take the first place as SR culprits (Chaudhry et al., 2012).

Urticaria was the most common entity induced by SNIUAA, followed by anaphylaxis and isolated angioedema. The high proportion of NSAIDs-induced anaphylaxis agree with other studies in the last years which have considered NSAIDs as one of the main causes of drug-induced anaphylaxis, surpassing betalactam antibiotics (Doña et al., 2011; Aun et al., 2014; Jares et al., 2015; Mota et al., 2018).

Through the analysis of the contribution of the specific NSAIDs groups to the different clinical entities, we found that arylpropionics are the most frequent triggers of reactions involving skin, as previously reported (Doña et al., 2011; Demir et al., 2015). Interestingly, ibuprofen was the most common NSAID inducing angioedema, as it has been published (Chaudhry et al., 2012). Pyrazolones were the major cause of anaphylaxis, followed by arylpropionic acid derivatives. These two groups of drugs have been shown to play a key role in inducing anaphylaxis in adults (Jares et al., 2015), although a clear differentiation between CRs and SRs was not established. In addition, both pyrazolones and arylpropionics appear to be relevant in children anaphylaxis in one study (Ensina et al., 2014); however, in such study most patients reacted to NSAIDs from different chemical groups.

Arylacetics were the third cause of anaphylaxis in our study. Diclofenac was also an important trigger of anaphylactic reactions in a study collecting 20-year-period data (van der Klauw et al., 1996), and similar findings have been reported by others (Picaud et al., 2014). However, to this day it is difficult to explain why certain drugs may induce more frequently some specific clinical manifestations.

In addition to anaphylaxis and skin involvement, we have also found that some patients with SNIUAA developed exclusively respiratory symptoms. As these patients tolerated ASA, we believe they may represent a new phenotype that should be considered, as reported (Doña et al., 2011; Corominas et al., 2012; Perez-Alzate et al., 2016). Nevertheless, although the prevalence of such phenotype does not seem to be very high, it needs to be established.

According to the ENDA, SRs to NSAIDs are classified into SNIUAA and SNIDR, depending on the time interval, with SNIUAA showing an acute onset (within the first hours), and SNIDR usually appearing 24 h after NSAIDs intake (Kowalski et al., 2013). The former are considered immediate reactions triggered by an IgE-mediated response, as supported by BAT and ST positivity (Schneider et al., 1987; Himly et al., 2003); notwithstanding, only few experimental studies have dealt with the quantification of IgE antibodies (Schneider et al., 1987; Blanca et al., 1989; Harrer et al., 2010). SNIDR are considered non-immediate reactions induced by a T cell-mediated response, as supported by positive delayed reading IDT and/or PTs to the culprit drug (Macias et al., 2007; Blanca-Lopez et al., 2016) and by T-cell infiltrates (Kowalski et al., 2013). However, controversies exist for reactions taking place in the time interval ranging from the first hours to 24 h after drug intake (Demoly et al., 2014). Indeed, this interval has not been clearly defined in the ENDA classification (Kowalski et al., 2013). In our study, some patients reported reactions within 1 to 6 h after drug intake. Nonetheless, when the latency period according to the clinical history is considered, the existence of some bias should not be excluded.

Although most SNIUAA patients in our study developed clinical symptoms within the first hour after drug intake, an important proportion did develop the reaction in a larger interval. The time interval between drug administration and the onset of the reaction may depend on the production of metabolites, which have not been identified for most drugs yet. The exception is represented by metamizole, which more than 20 metabolites already identified (Levy et al., 1995); however, few studies have analyzed their immunogenic potential (Schneider et al., 1987; Zhu et al., 1992; Ariza et al., 2014).

The diagnosis of SR patients is often complex, with the lack of *in vitro* tests and the low sensitivity of STs being the major drawbacks (Kowalski et al., 1999; Gomez et al., 2009; Blanca-Lopez et al., 2016). In our study, STs were positive in 23% of metamizole-induced SNIUAA, as observed in previous studies (Kowalski et al., 1999; Gomez et al., 2009; Blanca-Lopez et al., 2016). For SNIDR, positive results in STs were found in 68% of patients, although scarce information is available (Nettis et al., 2003; Andrade and Goncalo, 2011; Pinho et al., 2017). Therefore, the diagnosis frequently relies on DPT. This is not a risk-free procedure, requires trained personnel and specific resources, and is contraindicated in severe reactions (Doña et al., 2019). In addition, DPT may not be performed in mislabeled CR patients. In fact, we excluded 67 patients experiencing only one reaction and having negative STs (when performed) as they did not undergo DPT to the culprit due to the severity of the reported reaction. This may contribute to some bias in our study; however, such patients represent a small percentage from the total evaluated (1.17%).

An important number of patients were diagnosed based on repeated episodes to the drugs and ASA tolerance. This may also represent a limitation in our study, as diagnosis was not confirmed by DPT with the culprit. Nevertheless, in our population, patients with positive DPT to NSAIDs reproduced the recorded clinical symptoms, suggesting that their clinical entity was reliable. Previous studies looking at CRs established that at least 3 reported episodes were required for accurate diagnosis (Blanca-Lopez et al., 2013), in SRs we have considered that 2 unequivocal episodes were sufficient, providing that the clinical history was reliable (Blanca-Lopez et al., 2016).

Summarizing, we have reported the largest series of patients with SRs to NSAIDs studied to date, describing the drugs/groups of drugs involved in the different clinical entities, and the methods to achieve diagnosis. Since NSAIDs are highly consumed worldwide, the provided information may be of interest for evaluating other populations exposed to these drugs.

## Data Availability Statement

The datasets generated for this study are available on request to the corresponding authors.

## Ethics Statement

The studies involving human participants were reviewed and approved by Comité de Ética de la Investigación Provincial de Málaga. The patients/participants provided their written informed consent to participate in this study.

## Author Contributions

NP-S, ID, GB, MS, and AT recruited patients and performed the clinical evaluations. MT and JC-G contributed to study design. NP-S and ID wrote the first draft of the manuscript. MT and JC-G corrected the manuscript. All authors revised and approved the submitted version of the manuscript.

## Funding

This work was supported by grants co-funded by the European Regional Development Fund (ERDF), from the Carlos III National Health Institute (ARADyAL network RD16/0006/0001, and PI17/01593), and from the Sociedad Española de Alergología e Inmunología Clínica (SEAIC; Ref. Convocatoria Ayudas 2016, and Convocatoria Ayudas 2018 REF: 18B02).

## Conflict of Interest

The authors declare that the research was conducted in the absence of any commercial or financial relationships that could be construed as a potential conflict of interest.

The reviewer RC declared a past co-authorship with one of the authors ID to the handling editor.

## Abbreviations

ASA, acetylsalicylic acid; CR, cross-hypersensitivity reaction; DPT, provocation test; IDT, intradermal test; NSAIDS, non-steroidal anti-inflammatory drugs; PT, patch test; SPT, skin test; SNIDR, single-NSAID-induced delayed reaction; SNIUAA, single-NSAID-induced urticaria/angioedema or anaphylaxis; SR, selective reaction.
