# Supplementary material for: Evaluation of Subjects Experiencing Allergic Reactions to Non-Steroidal Anti-Inflammatory Drugs: Clinical Characteristics and Drugs Involved
Source: Front Pharmacol. 2020 Apr 21;11:503. doi: 10.3389/fphar.2020.00503 (PMC7212440; doi:10.3389/fphar.2020.00503)
Supplement: Supplementary file 1 [file DataSheet_1.docx]

**Supplementary Table S1.** Number of patients and pharmacological groups/drugs involved in SRs.

|  |  | **Total** (N=512), n (%) | **SNIUAA** (N=461),  n (%) | **SNIDR** (N=51),  n (%) | p |
| --- | --- | --- | --- | --- | --- |
| *Pyrazolones* |  | 223 (43.5) | 209 (45.3) | 14 (27.4) | 0.015 |
|  | Metamizole | 207 (40.4) | 193 (41.8) | 14 (27.4) | 0.047 |
|  | Propyphenazone | 16 (3.1) | 16 (3.4) | - | NA |
| *Propionic derivatives* |  | 164 (32) | 143 (31.1) | 21 (41.17) | 0.14 |
|  | Ibuprofen | 126 (24.6) | 122 (26.4) | 4 (7.8) | 0.003 |
|  | Naproxen | 16 (3.1) | 11 (2.4) | 5 (9.8) | 0.004 |
|  | Dexketoprofen | 9 (1.7) | 6 (1.3) | 3 (5.8) | 0.018 |
|  | Ketoprofen | 13 (2.5) | 4 (0.8) | 9 (17.6) | 4.9e-13 |
| *Paracetamol* |  | 41 (8) | 39 (8.4) | 2 (3.9) | 0.257 |
| *Arylacetic derivatives* |  | 48 (9.3) | 43 (9.3) | 5 (9.8) | 0.912 |
|  | Diclofenac | 44 (8.5) | 40 (8.6) | 4 (7.8) | 0.84 |
|  | Aceclofenac | 4 (0.8) | 3 (0.6) | 1 (1.9) | 0.344 |
| *ASA* |  | 21 (4.1) | 19 (4.1) | 2 (3.9) | 0.946 |
| *Oxicams* |  | 7 (1.4) | 5 (1.1) | 2 (3.9) | 0.098 |
|  | Lornoxicam | 1 ( 0.2) | 1 (0.2) | - | NA |
|  | Meloxicam | 1 (0.2) | 1 (0.2) | - | NA |
|  | Piroxicam | 5 (0.9) | 3 (0.6) | 2 (3.9) | 0.08 |
| *Etofenomate* |  | 5 (0.9) | 1 (0.2) | 4 (7.8) | 0.0004 |
| *Lisine clonixinate* |  | 1 (0.2) | 1 (0.2) | - | NA |
| *Celecoxib* |  | 1 (0.2) | - | 1 (1.9) | NA |
| *Nabumetone* |  | 1 (0.2) | 1 (0.2) | - | NA |

SNIDR, single-NSAID-induced delayed reactions; SNIUAA, single-NSAID-induced urticaria/angioedema or anaphylaxis; SRs, selective reactions; NA, not applicable.

**Supplementary Table S2.** Number of patients and clinical entities induced by each pharmacological group/drugs in SNIUAA. In patients reporting more than one episode, only the most severe one is recorded in this table. A total of 8 patients reported one episode of anaphylaxis and another episode of urticaria after metamizole intake, and one patient after ASA intake. Two patients reported one episode of urticaria and another one of AE induced by diclofenac. One patient reported one episode of asthma and another one of anaphylaxis after paracetamol, and one patient reported two episodes of urticaria after piroxicam and another one after meloxicam intake. Finally, one patient reported one episode of asthma and three of rhinitis after ibuprofen intake.

|  |  | **Anaphylaxis** (N=170),  n (%) | **AE** (N=86), n (%) | **Urticaria** (N=199),  n (%) | **Asthma** (N=4), n (%) | **Rhinitis** (N=2),  n (%) | p |
| --- | --- | --- | --- | --- | --- | --- | --- |
| *Pyrazolones* (n=209) |  | 120 (57.4) | 9 (4.3) | 79 (37.7) | 1 (0.4) | - | 2.2e-16 |
|  | Metamizole (n=193) | 112 (58) | 8 (4.1) | 72 (37.3) | 1 (0.5) | - | 2.2e-16 |
|  | Propyphenazone (n=16) | 8 (50) | 1 (6.2) | 7 (43.7) | - | - | 0.348 |
| *Propionic derivatives* (n=143) |  | 29 (20.2) | 57 (39.8) | 53 (37.06) | 2 (1.4) | 2 (1.4) | 4.8e-15 |
|  | Ibuprofen (n=122) | 24 (19.6) | 48 (39.3) | 46 (37.7) | 2 (1.6) | 2 (1.6) | 4.8e-12 |
|  | Naproxen (n=11) | 3 (27.3) | 5 (45.4) | 3 (27.3) | - | - | 0.094 |
|  | Dexketoprofen (n=6) | 2 (33.3) | 3 (50) | 1 (16.6) | - | - | 0.138 |
|  | Ketoprofen (n=4) | - | 1 (25) | 3 (75) | - | - | 1 |
| *Arylacetic derivatives* (n=43) |  | 11 (25.6) | 5 (11.6) | 27 (62.8) | - | - | 0.03 |
|  | Diclofenac (n=40) | 9 (22.5) | 5 (12.5) | 26 (65) | - | - | 0.018 |
|  | Aceclofenac (n=3) | 2 (66.6) | - | 1 (33.3) | - | - | 0.597 |
| *Paracetamol* (n=39) |  | 9 (23.1) | 4 (10.2) | 26 (66.6) | - | - | 0.01 |
| *ASA* (n=19) |  | 1 (5.2) | 7 (36.8) | 10 (52.6) | 1 (5.2) | - | 0.002 |
| *Oxicams* (n=5) |  | - | 4 (80) | 1 (20) | - | - | 0.03 |
|  | Piroxicam (n=3) | - | 2 (66.6) | 1 (33.3) | - | - | 0.217 |
|  | Lornoxicam (n=1) | - | 1 (100) | - | - | - | NA |
|  | Meloxicam (n=1) | - | 1 (100) | - | - | - | NA |
| *Etofenomate* (n=1) |  | - | - | 1 (100) | - | - | NA |
| *Lysine clonixinate* (n=1) |  | - | - | 1 (100) | - | - | NA |
| *Nabumetone* (n=1) |  | - | - | 1 (100) | - | - | NA |

AE, angioedema; ASA, acetylsalicylic acid; NA, not applicable.

**Supplementary Table S3.** Number of patients and clinical entities induced by each pharmacological group/specific drugs in SNIDR. One patient reported one episode of MPE after diclofenac intake and another episode after aceclofenac.

|  |  | **AE**  (N=1),  n (%) | **Urticaria**  (N=6),  n (%) | **FDE** (N=19),  n (%) | **MPE**  (N=18),  n (%) | **BE**  (N=2),  n (%) | **SJS/TEN**  (N=1),  n (%) | **CE** (N=4),  n (%) | p |
| --- | --- | --- | --- | --- | --- | --- | --- | --- | --- |
| *Pyrazolones* (n=14) |  | - | 1 (7.1) | 5 (35.7) | 7 (50) | - | 1 (7.1) | - | 0.369 |
|  | Metamizole  (n= 14) | - | 1 (7.1) | 5 (35.7) | 7 (50) | - | 1 (7.1) | - | 0.369 |
| *Propionic derivatives* (n=21) |  | 1 (4.7) | 2 (9.5) | 11 (52.3) | 4 (19) | 1 (4.7) | - | 2 (9.5) | 0.182 |
|  | Ibuprofen (n=4) | - | 1 (25) | 2 (50) | 1 (25) | - | - | - | 0.788 |
|  | Naproxen (n=5) | - | - | 4 (80) | 1 (20) | - | - | - | 0.339 |
|  | Dexketoprofen (n=3) | 1 (33.3) | - | - | 2 (66.6) | - | - | - | 0.158 |
|  | Ketoprofen (n=7) | - | 1 (14.3) | 5 (71.4) | - | 1 (14.3) | - | 2 (28.5) | 0.579 |
| *Arylacetic derivatives* (n=5) |  | - | - | 1 (20) | 3 (60) | - | - | 1 (20) | 0.291 |
|  | Diclofenac (n=4) | - | - | 1 (25) | 2 (50) | - | - | 1 (25) | 0.295 |
|  | Aceclofenac (n=1) | - | - | - | 1 (100) | - | - | - | NA |
| *Paracetamol* (n=2) |  | - | 1 (50) | 1 (50) | - | - | - | - | 0.43 |
| *ASA* (n=2) |  | - | - | - | 2 (100) | - | - | - | NA |
| *Oxicams* (n=2) |  | - | 1 (50) | - | - | 1 (50) | - | - | 0.464 |
|  | Piroxicam (n=2) | - | 1 (50) | - | - | 1 (50) | - | - | 0.464 |
| *Etofenomate* (n=4) |  | - | 1 (25) | 1 (25) | 1 (25) | - | - | 1 (25) | 0.333 |
| *Celecoxib* (n=1) |  | - | - | - | 1 (100) | - | - | - | NA |

AE, angioedema; BE, bullous exanthema; CE, contact eczema; FDE, fixed drug exanthema; MPE, maculopapular exanthema; SJS/TEN, Stevens-Johnson syndrome/toxic epidermal necrolysis; NA, not applicable.
